# Supplementary material for: Causal Relationship between Aging and Anorexia Nervosa: A White-Matter-Microstructure-Mediated Mendelian Randomization Analysis
Source: Biomedicines. 2024 Aug 16;12(8):1874. doi: 10.3390/biomedicines12081874 (PMC11351342; doi:10.3390/biomedicines12081874)
Supplement: Supplementary file 1 [file biomedicines-12-01874-s001.zip › Supplementary Figure/Figure S8/Leaveoneout plot of MR analysis for TL to ubm-b-1882.pdf]

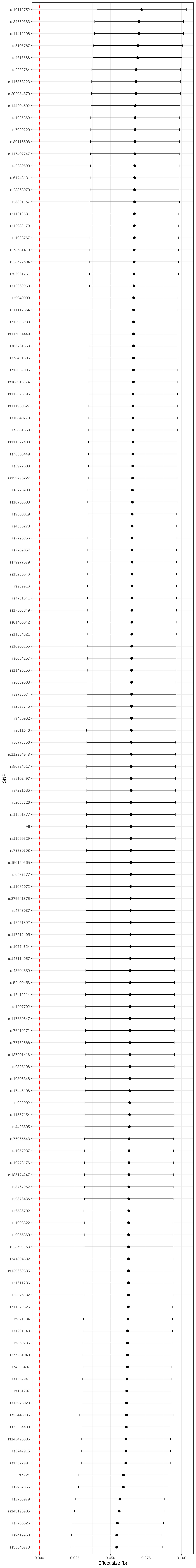
rs10112752
rs34550383
rs11412296
rs8105767
rs4616688
rs2282764
rs116863223
rs202034370
rs144204502
rs1985369
rs7099229
rs80116508
rs117407747
rs2230590
rs61748181
rs28363070
rs3891167
rs11212631
rs12932179
rs1023767
rs73581419
rs28577594
rs56061761
rs12369950
rs9940099
rs11117354
rs12925933
rs117034449
rs66731853
rs78491606
rs13062095
rs188918174
rs113525195
rs111950327
rs10840270
rs6881568
rs111527438
rs76666449
rs2977608
rs139795227
rs6790988
rs10768683
rs9600019
rs4530278
rs7790856
rs7209057
rs79977579
rs13230646
rs939916
rs4731541
rs17803849
rs61405042
rs11584821
rs10905255
rs6054257
rs11426156
rs6669563
rs3785074
rs2538745
rs450962
rs611646
rs6776756
rs112394943
rs80324517
rs8102497
rs7221585
rs2056726
rs11991877
All
rs11699829
rs73730598
rs150150565
rs6587577
rs11085072
rs376641875
rs4743037
rs12451892
rs117512405
rs10774624
rs145114957
rs45604339
rs59409453
rs12412214
rs1907702
rs117630647
rs76219171
rs77732866
rs137901416
rs9398196
rs10805346
rs17445108
rs932002
rs11557154
rs4498805
rs76065543
rs1957937
rs10773176
rs185174247
rs3767952
rs9878436
rs6536702
rs1003322
rs9955360
rs28502153
rs41304832
rs139669835
rs1611236
rs2276182
rs11579626
rs871134
rs1291143
rs869785
rs77231040
rs4695407
rs1332941
rs131797
rs16978028
rs35446936
rs75664430
rs142426306
rs5742915
rs17677991
rs4724
rs2967355
rs2763979
rs143190905
rs7705526
rs9419958
rs35640778
SNP
0.000
0.025
0.050
0.075
0.100
Effect size (b)
